# In vitro and in silico studies of the antiviral activity of polyhydrated fullerenes against influenza A (H1N1) virus

**DOI:** 10.1038/s41598-023-38128-3

**Published:** 2023-07-05

**Authors:** Polina Zaremba, Andrii Zaremba, Krystyna Naumenko, Mykhailo Yelipashev, Svitlana Zahorodnia

**Affiliations:** 1 Zabolotny Institute of Microbiology and Virology of NASU, 154 Acad. Zabolotny St., Kyiv, 03143 Ukraine; 2 Private Research Laboratory “Yelipashev”, 16 O. Davydova St., Kyiv, 02154 Ukraine

**Keywords:** Antivirals, Influenza virus

## Abstract

As of today, influenza viruses remain a relevant target for the development of antiviral compounds due to their rapid evolution and acquisition of the resistance to existing drugs. Fullerene derivatives have already shown the ability to successfully interact with viruses, and polyhydrated fullerenes (or fullerenols) are particularly attractive due to their compatibility with biological fluids and low toxicity. Therefore, the goal of this work was to study the effect of two batches of a mixture of polyhydrated fullerenes with a mass ratio of 78.1% C_60_/C_70_ and 21.9% C_76_/C_78_/C_84_ on the influenza A (H1N1) virus. It was determined that the mixture of fullerenols, along with the low toxicity, showed high antiviral activity with a decrease in the viral infectious titer up to 4 orders of magnitude. In addition, studied fullerenols did not affect the hemagglutination process and did not show any significant prophylactic activity. With the help of molecular docking and molecular dynamics simulation, the likely target of fullerenols' action was determined—the binding site of the RNA primer of the viral RNA-dependent RNA polymerase. Therefore, we assume that the high antiviral effect of polyhydrated fullerenes on influenza A virus is related to their interaction with the viral RNA polymerase.

## Introduction

Humanity has "officially" coexisted with influenza viruses for more than a century and, most likely, much longer. Since 1918, five pandemics have already been registered, three of them were caused by influenza A (H1N1) virus^1^. It has firmly established itself as an annual cause of acute respiratory viral infections and is especially dangerous for certain vulnerable categories of the population, such as elderly people over 65 years old, immunocompromised individuals, pregnant women, children, and people with chronic diseases. Antiviral drugs are very necessary for this part of the world's population. Thus, the root of the problem is that influenza viruses quickly mutate and acquire resistance to drugs due to the peculiarities of their structure and, as a result, the phenomena of the antigenic drift and the reassortment^2^. To date, we have already abandoned the use of the adamantanes (inhibitors of the viral proton pump M2) due to the high resistance of influenza A virus strains (more than 69% of H1 subtypes)^3^. Currently, neuraminidase inhibitors are almost the only globally widespread type of anti-influenza drugs, of which oseltamivir and zanamivir are the most often used, including usage in Ukraine. Resistance of the H1N1 strain to oseltamivir increased dramatically before the start of the 2009 pandemic, although H1N1 pdm09 itself was mostly (> 98.5%) susceptible to neuraminidase inhibitors. As of today, influenza A virus remains susceptible to oseltamivir and zanamivir, but their use increases a frequency of mutations responsible for the resistance^4^. Therefore, the search for new drugs is constantly ongoing, but due to the complex and long-term development process, their number grows very slowly. Baloxavir marboxil, a cap-dependent endonuclease inhibitor of influenza virus, was the last to enter the pharmaceutical market, but it was approved for treatment and prevention only in people over 12 years of age^5^. Moreover, the situation with SARS-CoV-2 and a phenomenon of its co-infection with influenza viruses acutely demonstrate the need for antiviral agents with different mechanisms of action.

The need for safe drugs that can be used for all vulnerable categories remains, and recently scientists have paid more attention to nanomaterials, which have found wide application not only in the medical field. Unique carbon nanostructures—fullerenes—attract the attention of many scientists and specialists, primarily because of their chemical and physical properties, such as size, symmetry, stability, three-dimensionality and electronic configurations. As of today, there are already confirmations of the effect of fullerenes and their derivatives on HIV^6^, hepatitis C virus^7^, influenza A virus^8^, herpesviruses^9^, as well as an in silico studies of the effect on SARS-CoV-2^10^.

The main problem of using fullerenes in biology and medicine is high hydrophobicity, and, as a result, very low solubility in water^11^. However, this problem is solved by modifications of the molecule. To date, hydrated fullerenes or fullerenols with the general formula C_n_(OH)_x_ are sufficiently well studied^12^. The formation of soluble fullerenes makes it possible to combine them with liquids, including physiological ones (blood, lymph), which expands the range of applications^13^. In addition, hydrated fullerenes are stable and have relatively low toxicity, which undoubtedly makes them attractive candidates for the role of drugs, in particular, antiviral agents. That is why the aim of our work was to study the effect of two forms of the mixture of polyhydrated fullerenes K050 and K400 on the influenza A (H1N1) virus.

## Results

Based on the results obtained after the MTT assay, CC50 indicators (50% cytotoxic concentration) were calculated. For HyFn400 and for the reference-drug oseltamivir the indicators were 11.79 ± 0.58 mg/ml and 0.29 ± 0.01 mg/ml, respectively. HyFn050 at its maximum concentration of 2.5 mg/ml did not show any toxic effect on cells. Accordingly, due to the absence of toxicity it was impossible to calculate CC50 for the latter.

### Antiviral activity and determination of infectious titer de novo

**Figure 1:**
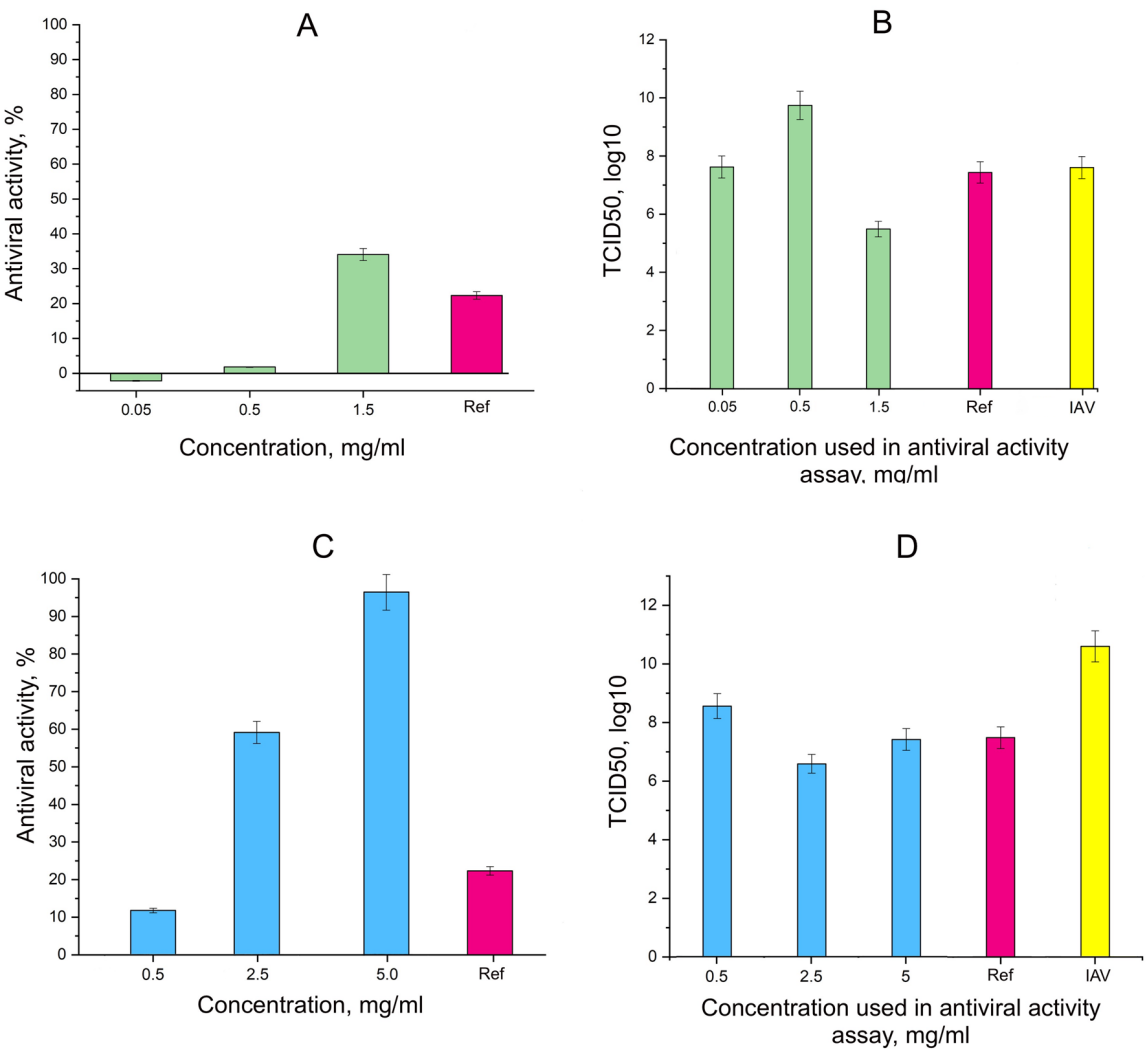
The effect of hydrated fullerenes on the reproduction process of influenza A (H1N1) virus and its infectious titer: (**A**) The antiviral activity of HyFn050 according to the post-exposure administration scheme; (**B**) The value of infectious titers de novo in TCID50 (log10) in the supernatant after determining the antiviral activity of HyFn050; (**C**) The antiviral activity of HyFn400 according to the post-exposure administration scheme; (**D**) The value of infectious titers de novo in TCID50 (log10) in the supernatant after determining the antiviral activity of HyFn400; *Ref* oseltamivir phosphate; *IAV *virus control.

The results of the calculation of the antiviral activity showed a clear dose-dependent inhibitory effect for the studied polyhydrated fullerenes (Fig. 1A,C). We observed the greatest activity at the highest tested concentrations: HyFn050 (1.5 mg/ml) inhibited H1N1 reproduction by 34.07 ± 1.70%, and HyFn400 (5 mg/ml) by 96.48 ± 4.82%. Oseltamivir at a concentration of 0.15 mg/ml showed the result of 22.33 ± 1.11% antiviral activity.

Determination of the infectious titer of the virus after the antiviral activity study of the fullerenols (that is, in the cell supernatant) showed its decrease in general by 2–4 orders of magnitude. The HyFn400 mixture showed a decrease in the infectious titer at all tested concentrations (Fig. 1D). The greatest drop in the infectious titer by 4.02 orders of magnitude was observed at a concentration of 2.5 mg/ml compared to the virus control (TCID50 = 10^10.61^). HyFn050 showed an effect on the TCID50 value only at the highest tested concentration of 1.5 mg/ml: a decrease of 2.13 orders of magnitude at the TCID50 of the virus control of 10^7.65^ (Fig. 1B). The activity of oseltamivir at a concentration of 0.15 mg/ml was manifested in a decrease in the infectious titer of the virus by 3.14 orders of magnitude compared to the control (TCID50 = 10^10.61^) (Fig. 1D).

### Prophylactic activity and determination of infectious titer de novo

**Figure 2:**
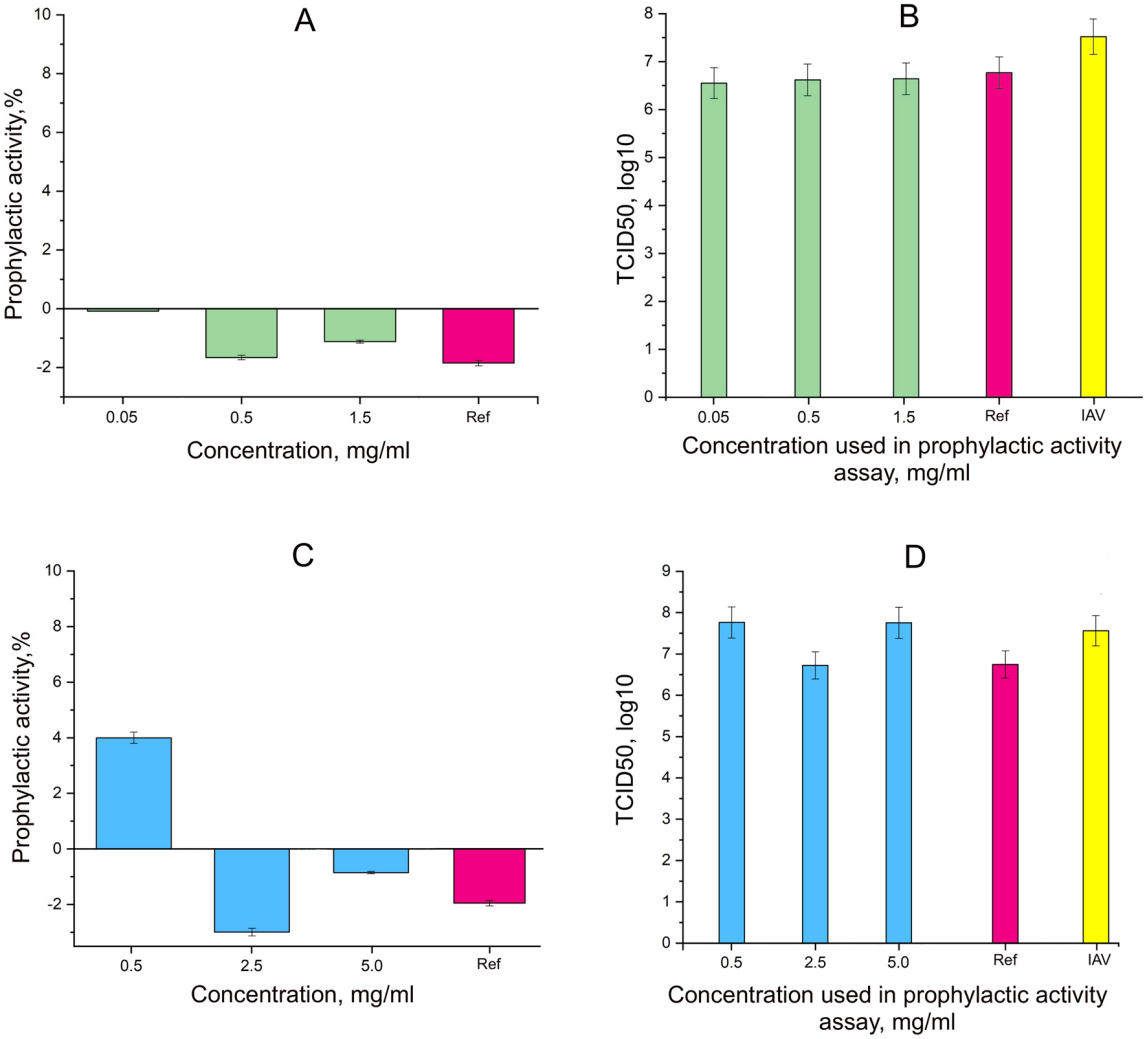
The prophylactic effect of hydrated fullerenes on the process of influenza virus infection: (**A**) Indicators of the prophylactic activity for HyFn050; (**B**) The value of infectious titers de novo in the supernatant after determining the prophylactic effect of HyFn050; (**C**) Indicators of the prophylactic activity for HyFn400; (**D**) The value of infectious titers de novo in the supernatant after determining the prophylactic effect of HyFn400; *Ref* oseltamivir phosphate; *IAV* virus control.

During 24 h of incubation with cells before influenza infection, hydrated fullerenes did not show high prophylactic activity. HyFn050 and oseltamivir did not affect the inhibition of the infection process at all (Fig. 2A). Fullerenols HyFn400 demonstrated a slight prophylactic activity at the lowest tested concentration of 0.5 mg/ml with the result of 4.00 ± 0.20% (Fig. 2C).

The calculation of the infectious titer after the prophylactic activity study of the compounds showed the following effect of fullerenols. HyFn0400 showed a slight decrease in the TCID50 value by 0.89 orders of magnitude compared to the virus control (TCID50 = 10^7.59^) at a concentration of 2.5 mg/ml (Fig. 2D). Similar activity was observed for HyFn050: in the concentration range of 0.05–1.5 mg/ml, the infectious titer decreased by an average of 0.9 orders of magnitude, compared to the virus control (TCID50 = 10^7.52^) (Fig. 2B). And oseltamivir at a concentration of 0.15 mg/ml showed a decrease in titer by 0.75 orders of magnitude (Fig. 2B).

### Hemagglutination inhibition reaction

Previously, before the HIR (hemagglutination inhibition reaction) with hydrated fullerenes, the titer of the virus was determined using the hemagglutination reaction, which was 64 HAU/25 μl. The ability of the studied structures to directly affect erythrocytes in the concentration range of 0.01–10 mg/ml for HyFn400, 0.01–1.25 mg/ml for HyFn050 and 0.015–0.15 mg/ml for oseltamivir was also tested. Neither compound was found to cause agglutination of the erythrocytes. The HIR was carried out in the same range of concentrations. The results showed that hydrated fullerenes and oseltamivir do not affect the ability of the influenza A (H1N1) virus to agglutinate guinea pig erythrocytes.

### Molecular docking and molecular dynamics simulation

**Figure 3:**
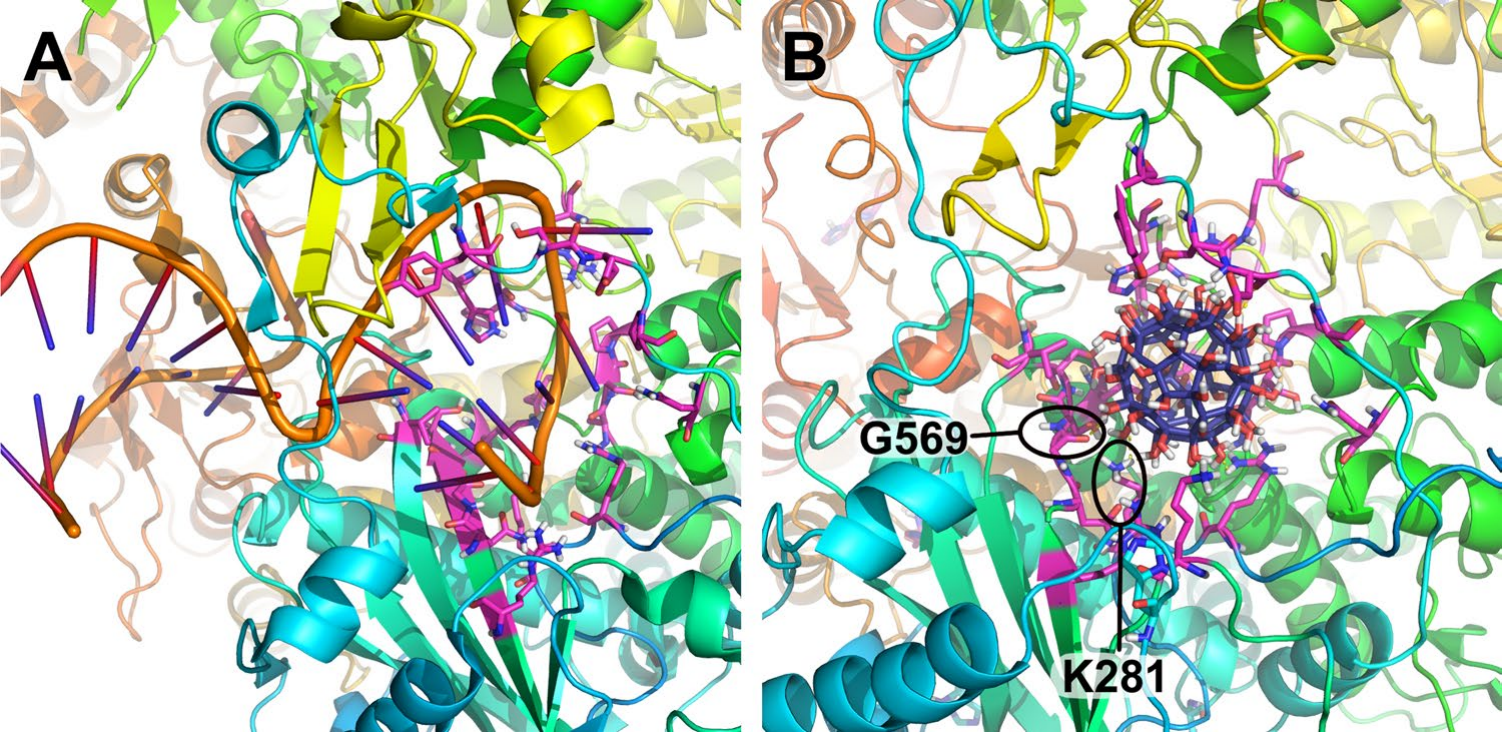
The RdRp of H1N1 in complex with primer RNA (**A**) and C_60_(OH)_60_ (**B**). Amino acids that directly surround a fullerenol during its interaction with viral polymerase are highlighted in pink. It can be clearly seen that the same amino acids are directly involved in the interaction of H1N1 RdRp with vRNA.

According to the molecular docking, the RdRp/fullerenol complex, where the latter partially covered the site of interaction of the primer viral RNA (vRNA) was characterized by the best evaluation score (− 6.7 kcal/mol). In Fig. 3B amino acids that directly interact with C_60_(OH)_60_ are highlighted in pink: R279, K281, Y393, S395, D396, P398, V517, R566, N568, G569, T570, S571, K574, A651, S652, P653, N696—PA (polymerase acidic protein); D27, Y30, H32, R238—PB1 (polymerase basic protein 1). In Fig. 3A, these amino acids are also highlighted. Together with others, they participate in the interaction of the hook structure of the 5′-promoter with RdRp.

**Figure 4:**
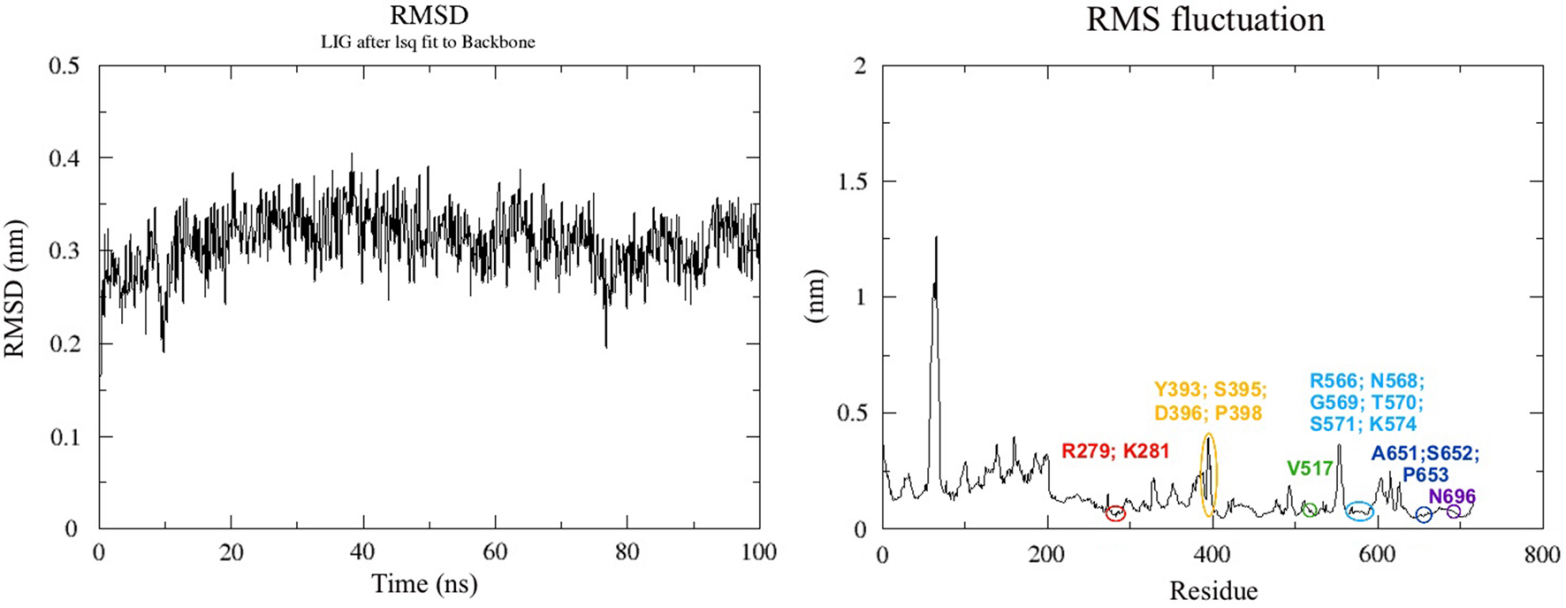
The root-mean-square deviation of the ligand-receptor complex and the root-mean-square fluctuation of RdRp PA subunit. At the beginning of the simulation, the average RMSD value of C_60_(OH)_60_ quickly reached its maximum at ~ 3 Å, and after that stabilized. And the mentioned value remained until the end of the simulation with the exception of minor oscillations. At the same time, the amplitude of the RMSD of fullerenol is ~ 1 Å. The RMSF of RdRp PA showed a high degree of stability of most of the amino acids involved in the interaction with C_60_(OH)_60_. Residues Y393, S395, D396, and P398 are exceptions because they are a part of the highly labile disordered loop.

The molecular dynamics of polyhydrated C_60_ in the complex with H1N1 RdRp was relatively homogeneous and was characterized by a stable preservation of the ligand position throughout the simulation period. The root-mean-square deviation (RMSD) of C_60_(OH)_60_ during the first 20 ns of the simulation stabilized at a value of ~ 3 Å and remained until the end of the simulation with the exception of minor fluctuations (Fig. 4). The range of RMSD fluctuations was about 1 Å. That, together with the constant preservation of the general level of RMSD, may indicate the sufficient stability of the studied ligand in the complex with RdRp.

The root-mean-square fluctuation (RMSF) distribution of amino acids of the RdRp PA subunit showed mostly its minimum value for amino acids involved in the interaction with polyhydrated C_60_. Only Y393, S395, D396, and P398 had a high RMSF. However, it should be noted that these amino acids are a part of the disordered and labile loop that directly interacts with the solvent during simulation.

**Figure 5:**
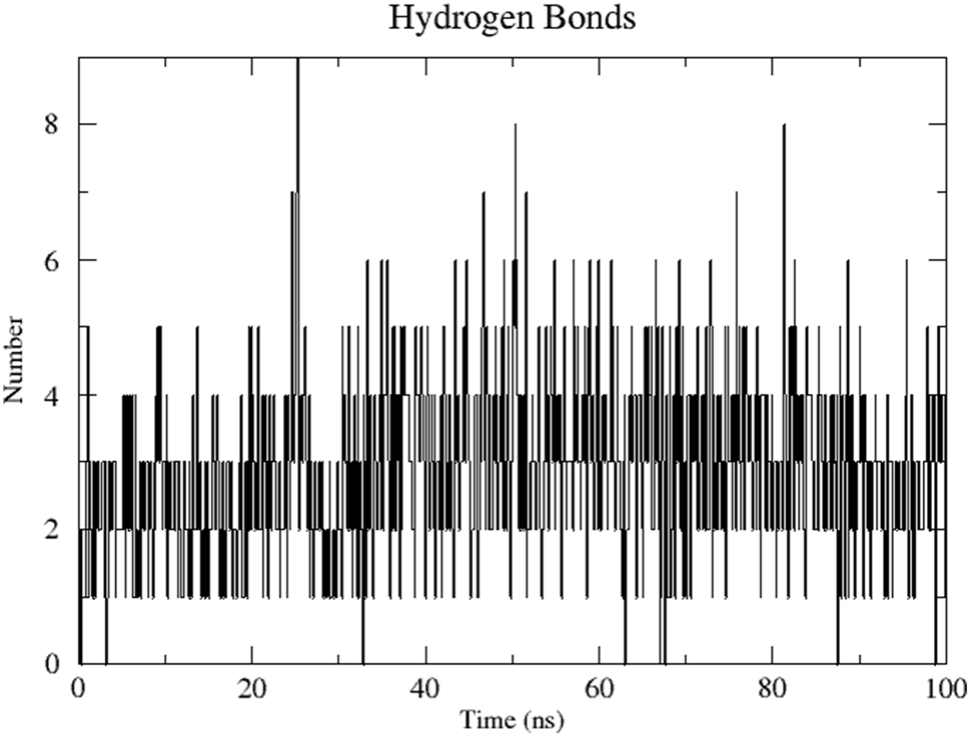
The number of hydrogen bonds between C_60_(OH)_60_ and RdRp. On average, it varies between 2–3.

Among the main orienting interactions, hydrogen bonds of C_60_(OH)_60_ with amino group K281 and carbonyl G569 (which are a part of PA subunit) should be highlighted. These connections are stable enough to be observed throughout the simulation. Additional hydrogen bonds with: R279, S395, D396, R566, G569, T570 and S571—PA, H32 and R238—PB1 were also present in some periods of the simulation. In general, the number of hydrogen bonds between the ligand and the receptor ranged from 2 to 3 during the simulation (Fig. 5) and after some rare jumps up to 6–7.

The value of the interaction enthalpy between C_60_(OH)_60_ and RdRp, calculated on the basis of the molecular modeling trajectory, was sufficiently significant and equaled to − 129.55 ± 14.52 kcal/mol. That indicates a potentially high gain of the system in case of successful formation of the RdRp/C_60_(OH)_60_ complex. Moreover, the contribution of both van der Waals and electrostatic energy to the ligand-receptor interaction were close and provided the basis for the stability of the formed associate: − 61.88 ± 7.62 kcal/mol and − 58.59 ± 18.71 kcal/mol, respectively. A much smaller contribution was provided by solvation free energy – 9.08 kcal/mol. In Table 1, it is represented by polar (− 0.58 ± 19.65 kcal/mol) and non-polar (-8.5 ± 1.14 kcal/mol) components. According to the fact that the entropy penalty was equal to 55.85 ± 0.25 kcal/mol, the final free energy of formation of the C60(OH)60/RdRp complex was − 73.7 ± 14.52 kcal/mol.

**Table 1:** Energy components of the final free energy of interaction.

	E_vdw_	E_el_	E_gb_	E_sasa_	ΔH	-TΔS	ΔG
Average	− 61.88	− 58.59	− 0.58	− 8.5	− 129.55	55.85	− 73.7
SD	7.62	18.71	19.65	1.14	14.52	0.25	14.52

## Discussion

Studies of the activity of polyhydrated fullerenes against influenza A (H1N1) virus indicate their ability to influence the process of the virus reproduction. Considering the lack of interaction with the hemagglutinin according to the results of the hemagglutination inhibition reaction, we assume that HyFn050 and HyFn400 do not interact directly with surface structures of the virus particle. Also, under the prophylactic scheme of the study, mixtures of fullerenols did not show a significant activity with a decrease in the infectious titer by less than 1 order of magnitude. Considering this, we believe that the studied pohydrated fullerenes do not have an immunomodulatory effect at the cellular level similar to interferon-inducing antiviral compounds^14^.

Hydrated fullerenes HyFn050 and HyFn400 demonstrated the greatest effect on influenza virus reproduction after the establishment of infection in MDCK cells. We assume that a high dose-dependent antiviral effect, together with a decrease in the infectious titer up to 4 orders of magnitude, may be due to the interaction of fullerenes with influenza structures during the reproduction of the latter in the cell.

Some studies indirectly testify to the significant role of charge distribution on the surface of a fullerene in this process. In particular, Kataoka et al.^7^ demonstrated the ability of fullerene derivatives to inhibit RNA polymerase and protease of hepatitis C virus. At the same time, the C_60_ fullerene derivative which contained an additional carboxyl group showed three times greater activity against polymerase compared to protease. And Shoji et al.^8^ proved the ability of fullerene derivatives to inhibit the activity of the PA subunit of RdRp of influenza A H1N1 and H3N2 in their study. In this case, derivatives of C_60_ with a small negatively charged substituent on the surface showed a stronger inhibitory effect compared to derivatives with more massive and/or positively charged substituents. Therefore, we can assume that the presence of a small negatively charged substituent on the surface of C_60_ fullerenes may be an important factor for effective inhibition of viral RNA polymerases. The mixture studied in our work (C_60_/C_70_/C_76_/C_78_/C_84_)(OH)_40_ contains fullerenes of different sizes, but with a similarly hydrated surface. Each of the carbon atoms was bonded to one hydroxyl group and three other carbon atoms. Thus, the polyhydrated fullerenes that appear in our work had a relatively homogeneous surface lined with highly electronegative oxygen atoms. Taking into account the above-mentioned studies and the results obtained by us, we assume that the drug forms of fullerenols K050 and K400, represented by two batches HyFn050 and HyFn400, can have an inhibitory effect on the activity of the RdRp of the influenza A virus. In addition, the high hydrophilicity of the surface makes it difficult for fullerenols to pass through the cell membrane, which explains the activity at high concentrations and the dose-dependent effect.

In order to understand the direct mechanisms underlying the interaction between the RdRp of influenza A virus and the investigated hydrated fullerenes, molecular docking was performed. According to its results it was determined that the most likely site of fullerenol binding is the pocket of RdRp interaction with the RNA primer, more precisely, with the hook structure of its 5′-end^15^. In our opinion, this is logical because the charge distribution on the nucleic acid surface can be considered similar to the charge distribution on the surface of hydrated fullerenes. A further simulation study confirmed the high affinity of C_60_(OH)_60_ to the previously identified pocket. The RMSD of the ligand and the drop in the free energy of the system are the first evidence. The RMSD of hydrated C_60_ quickly stabilizes within ~ 3 Å (range of oscillations ~ 1 Å). Which, according to modern data, is included in the RMSD optimum for a ligand in a complex with its potential receptor^16^. At the same time, the significantly negative value of the binding free energy (− 73.7 ± 14.52 kcal/mol) indicates the high stability of the RdRp/C_60_(OH)_60_ complex. This is also confirmed by the low RMSF value characteristic of RdRp PA amino acids interacting with the ligand. The only exceptions are amino acids Y393, S395, D396 and P398. However, they are included in the highly labile disordered part of the PA-subunit, which, moreover, directly interacts with the solvent. Therefore, they can be considered as an exception in this case.

The main forces stabilizing the studied complex are hydrogen bonds, as well as a steric compatibility. This is reflected in the calculated high values of both van der Waals interactions and electrostatic energy as the main components of the enthalpy of the system. The interaction of C_60_(OH)_60_ with PA K281 and G569 was the most stable: all analyzed frames of the trajectory contained them. The formation of additional interactions with many of the above-described amino acid residues was also observed locally. Thus, the average number of hydrogen bonds fluctuated around 2–3, although significant excesses of this number were also observed.

In general, the placement of C_60_(OH)_60_ in the binding site of the 5′-end of the RNA primer, as well as its affinity to this site, in our opinion, may be related to its previously determined biological properties.

## Conclusions

The studied batches of the mixture of polyhydrated fullerenes K050 and K400 have an antiviral effect against influenza A (H1N1) virus, which is manifested in the suppression of viral reproduction by a maximum of > 94% and a decrease in the infectious titer de novo by 4 orders of magnitude. Since in the simulation experiment C_60_(OH)_60_ demonstrated a significant level of affinity to RNA-dependent RNA polymerase, in particular to the binding site of the RNA primer, we assume that the described effect is due to the interaction of the studied structures with the viral RNA polymerase. Therefore, we believe that this drug is promising for further research as an antiviral agent.

## Materials and methods

### Materials

MDCK cell culture and influenza A (H1N1) virus strain A/FM/1/47 were used for the study. Both were obtained from the L.V. Gromashevsky Institute of Epidemiology and Infectious Diseases of the National Academy of Medical Sciences of Ukraine.

The drug of Ukrainian production in two forms K050 and K400 ((C_60_/C_70_/C_76_/C_78_/C_84_)(OH)_40_) is an aqueous colloidal solution of a polyhydrated mixture of fullerenes with a mass ratio of 78.1% C_60_/C_70_ and 21.9% C_76_/C_78_/C_84_. The forms of the drug differ in the initial concentration. Two batches with different concentrations were tested: HyFn050 2.5 mg/ml (FN1120CSMP4K050) and HyFn400 20 mg/ml (FN1120CSMP5K400). Oseltamivir phosphate (Macleods Pharmaceuticals LTD) was used as a reference drug.

### Cultivation of MDCK

MDCK cell culture was grown in sterile flasks (Sarstedt, Germany) on a growth medium: 46% DMEM (Sigma, USA), 46% RPMI 1640 (Sigma, USA), 8% fetal bovine serum inactivated by heating at 56 °C (Sigma, USA) and 10 μl/ml penicillin streptomycin solution (Biowest, France). Cells were subcultivated at ratio of 1:10 every 48 h. For this 0.025% Trypsin–EDTA solution (Biowest, France) and 0.02% Versene solution (Biowest, France) were used, then cells were resuspended in growth medium. Cells were incubated in a thermostat at 37 °C in an atmosphere of 5% CO2.

### Determination of cytotoxicity

The cytotoxicity was determined using the MTT assay. The supernatant was removed from a 96-well plate (SPL Life Sciences, Republic of Korea) with a monolayer of MDCK cells and the test compounds diluted in the growth medium were added to the wells in the concentration range from 5 to 20 mg/ml for HyFn400, from 0.25 to 2.5 mg/ml for HyFn050, and from 0.01 to 1 mg/ml for oseltamivir. The compounds were incubated together with the cells for 48 h in a thermostat at 37 °C in an atmosphere of 5% CO_2_. After that MTT (3-(4,5-dimethylthiazol-2-yl)-2,5-diphenyltetrazolium bromide, neoFroxx, Germany) was dissolved in the sterile phosphate-buffered saline (PBS, pH = 7.4) to the concentration of 5 mg/ml and added to the wells in volume of 20 μl. The plate was incubated for 3 h and, after removing the supernatant, 150 μl of 96% ethyl alcohol was added. The optical density was measured on a Multiskan FC reader (Thermo Scientific, Waltham, MA, USA) at the wavelength of 538 nm.

### Antiviral activity

For a post-exposure scheme of introduction a 96-well plate (SPL Life Sciences, Republic of Korea) with a monolayer of cells was washed with 150 μl of PBS (pH = 7.4). 50 μl of virus suspension (TCID50/ml = 10^8,84^) in PRMI 1640 medium (Sigma, USA) was added to the washed wells and incubated for 90 min in a thermostat at 37 °C in an atmosphere of 5% CO2. After that, the supernatant was removed from the wells and 200 μl of compounds diluted in the supporting medium were added: 49% DMEM (Sigma, USA), 49% RPMI 1640 (Sigma, USA), 2% fetal bovine serum (Sigma, USA) and 1 μl/ml TPCK-treated trypsin (Sigma, USA). 200 μl of the supporting medium was added to the wells with the controls. The plate was incubated for 48 h at 37 °C and 5% CO2. After detection of cytopathic effect (CPE, granular destruction of the monolayer) the supernatant was removed, 50 μl of crystal violet dye (neoFroxx, Germany) 5 mg/ml in 19.2% ethanol 96% solution was added and incubated on a shaker for 10 min. After washing, the plate was left for 24 h at a room temperature to dry, then 150 μl of 96% ethanol was added. The optical density was measured at the wavelength of 538 nm on a Multiskan FC reader (Thermo Scientific, Waltham, MA, USA). The indicator of the antiviral activity of compounds (%) was calculated according to the following formula:

1 $${\text{Antiviral activity }} = \, \left( {{\text{A}} - {\text{B}}} \right)/\left( {{\text{C}} - {\text{B}}} \right)*{1}00\%$$

where A—an optical density of a studied concentration of a compound, B—an optical density of a control of a virus, C—an optical density of a control of the cells.

### Prophylactic activity

First, 50 μl/well of the studied compounds, diluted in a growth medium (46% DMEM (Sigma, USA), 46% RPMI 1640 (Sigma, USA), 8% fetal bovine serum (Sigma, USA) and 10 μl/ml penicillin streptomycin solution (Biowest, France), were added to the monolayer of cells in a 96-well plate (SPL Life Sciences, Republic of Korea) and left for incubation at 37 °C and 5% CO_2_ for 24 h. Next, the plate was washed with PBS (pH = 7.4) and 50 μl of virus suspension was added and incubated for 90 min (37 °C, 5% CO2). After that, the supernatant was removed from the wells and 200 μl of the supporting medium was added (49% DMEM (Sigma, USA), 49% RPMI 1640 (Sigma, USA), 2% fetal bovine serum (Sigma, USA) and 1 μl/ml TPCK-treated trypsin (Sigma, USA)). After 48 h of incubation (37 °C, 5% CO_2_), the presence of CPE was checked using an inverted microscope and cells were stained with crystal violet (neoFroxx, Germany). The value of the optical density was measured at the wavelength of 538 nm on a Multiskan FC reader (Thermo Scientific, Waltham, MA, USA). Based on the obtained results, the Prophylactic activity indicator was calculated (1).

### Determination of infectious titer de novo

To determine the infectious titer we used supernatant with the virus after 48 h of incubation from the antiviral and the prophylactic activity assays. A tenfold titration of the supernatant was performed using RPMI medium (Sigma, USA). Next, 50 μl of the suspension was added to the monolayer of cells in a 96-well plate (pre-washed with 150 μl of PBS, pH = 7.4). The plate was incubated for 90 min in a thermostat at 37 °C in an atmosphere of 5% CO_2_. After that, the supernatant was removed and 200 μl of the supporting medium (49% DMEM (Sigma, USA), 49% RPMI 1640 (Sigma, USA), 2% fetal bovine serum (Sigma, USA) and 1 μl/ml TPCK-treated trypsin (Sigma, USA)) was added. After the incubation for 48 h at 37 °C and 5% CO_2_, CPE was checked using an inverted microscope and cells were stained with crystal violet (neoFroxx, Germany). The value of the optical density was measured at the wavelength of 538 nm on a Multiskan FC reader (Thermo Scientific, Waltham, MA, USA). Based on the obtained data, TCID50 (Median Tissue Culture Infectious Dose) values were calculated according to the Reed-Muench method^17^.

### Hemagglutination reaction

The hemagglutination reaction was carried out using a 2% suspension of guinea pig erythrocytes in PBS (pH = 7.4) according to the standard method^18^.

Preparation of erythrocyte suspension: the animal's blood (2–5 ml) was taken postmortem into a test tube with anticoagulant—3.2% tri-sodium citrate dihydrate (Sigma, USA) diluted in sterile water (9 parts of blood + 1 part of anticoagulant). Carbon dioxide was used for euthanasia of the animals, which complies with the American Veterinary Medical Association. PBS (pH = 7.4) was added to the blood-anticoagulant mixture to a general volume of 10–12 ml. The test tube was centrifuged for 10 min at 1400 g and the supernatant was discarded. Centrifugation was repeated 2–3 times until the supernatant was clear. The resulting sediment of erythrocytes was diluted to the 2% suspension in PBS (pH = 7.4). Before use it was checked for autoagglutination: 25 μl PBS + 25 μl suspension in a plate with U-shaped wells left at a room temperature for 60 min.

All experimental protocols were approved by the Bioethics Commision of the Zabolotny Institute of Microbiology and Virology of the National academy of Sciences of Ukraine. The conducted experiments do not contradict the current legislation of Ukraine (Article 26 of the Law of Ukraine 5456-VI of 16.10.2012 “On protection of animals from cruel treatment”) and “General ethical principles of animal experiments”, adopted by the First National Congress on Bioethics (Kyiv, 2001) and corresponded to international bioethical standards. All methods are reported in accordance with ARRIVE guidelines.

### Hemagglutination inhibition reaction

The experiment was conducted according to a modified classical HIR^19^. The influenza virus dilution corresponding to 8 HAU/25 μl (hemagglutination units) was used for performing HIR. Reaction was carried out using a 2% suspension of guinea pig erythrocytes in PBS (pH = 7.4) in a serological 96-well plate with U-shaped wells (SPL Life Sciences, Republic of Korea). 25 μl of PBS (pH = 7.4) was added to the wells. 25 μl of the test compound was added to the first well and 25 μl was transferred to the next one (twofold titration). 25 μl was removed from the last well. 25 μl of virus was added to each well. 25 μl of the erythrocyte suspension was added to all wells. 25 μl of PBS (pH = 7.4) + 25 μl of the suspension were added to the control wells of the quality of erythrocytes. To control the effect of the studied compounds on erythrocytes, 25 μl of virus-free suspension was added to the corresponding dilutions of the substances. It was mixed on a shaker for 1 min. The result was taken into account after 60 min of incubation at a room temperature.

### Preparation of input data for the in silico studies

The selected file of the 3D structure of H1N1 RNA-dependent RNA polymerase (RdRp) (PDB ID: 7NK1), due to the lack of coordinate data for some amino acids, was completed based on the template using the SWISS-MODEL online resource^20^. For the further studies data of all molecules except the structure of the RdRp heterotrimer were deleted. The structure of the ligand – C_60_ fullerenol was generated using the Avogadro molecular editor^21^.

### Molecular docking

The molecular docking of fullerenol C_60_(OH)_60_ with H1N1 RdRp was performed using the Vina Wizard tool of the PyRx software package^22^. For this study, the grid box included the entire heterotrimeric protein simultaneously. The value of exhaustiveness due to the predicted large protein–ligand interaction site was 40. For further simulation experiments, the position of fullerenol with the best evaluation score was selected.

### Simulation of molecular dynamics

The actual construction of the system was carried out by the CHARMM-GUI toolkit^23^ using the CHARMM36m force field^24^ and involved the creation of a rectangular box with periodic boundary conditions, which contained the RdRp/fullerenol complex, water as a solvent (TIP3P model), K^+^ and Cl^−^ ions at the physiological concentration 0.15 M. After construction, the system underwent the steepest descent minimization to a system energy value of less than 1000 kJ/mol/nm (5000 steps). Next, the equilibration was carried out under conditions of stable volume and temperature of the system (classical NVT, Nose–Hoover thermostat, T-coupling constant of 1 ps, Van-der-Waals with Lennard–Jones potential and a 1.2 nm cut-off, electrostatic with a 1.2 nm cut-off, LINCS algorithm for covalent bonds, 303.15 K, the time step – 2 fs and the constraints for the protein) for 125 ps. The main simulation differed from the equilibration stage only by the additional condition of Parrinello-Raman barostat at 1 atm and the absence of restrictions on the protein position. The system temperature was 303.15 K. The equilibration and molecular simulations (100 ns) were performed in GROMACS 2020.6^25^ using GPU as an accelerator.

### Calculation of the binding free energy

The gmx_MMPBSA 1.5.2 package was used to calculate the free energy using the MM/GBSA (Molecular Mechanics/Generalized Born Surface Area) method^26^. The source of input data was the trajectory (the last 80% of frames – 800 in total) generated by GROMACS in the process of the molecular dynamics simulation of target associates. The polar contribution of solvation was estimated on the basis of the GB-OBC2 model^27^, and the non-polar contribution was estimated on the basis of SASA (solvent-accessible surface area). The contribution of the entropy component was calculated by the IE (interaction entropy) method^28^.

### Analysis of the results obtained in silico

The results were analyzed using standard software provided by GROMACS (trjconv, rms, rmsf, hbond) and gmx_MMPBSA (gmx_MMPBSA_ana). Visualization was performed using XMGRACE^29^ and Pymol 1.8^30^.

### Statistical processing of data obtained in vitro

Each experiment was carried out in three independent repetitions of 3 wells for 1 concentration of the compound. Statistical data processing was performed in the Origin 2018 program. According to standard approaches standard deviation was calculated and confidence interval was determined (*p *≤ 0.05 for all arrays).

## Data availability

All data generated or analysed during this study are included in this published article.

## Abbreviations

CC50: 50% cytotoxic concentration
TCID50: Median tissue culture infectious dose
IAV: Influenza A virus
HIR: Hemagglutination inhibition reaction
RdRp: RNA-dependent RNA polymerase
RMSD: Root mean square deviation
RMSF: Root-mean-square fluctuation
